# Age-related gene expression analysis in enteric ganglia of human colon after laser microdissection

**DOI:** 10.3389/fnagi.2014.00276

**Published:** 2014-10-15

**Authors:** Susan Hetz, Ali Acikgoez, Corinna Moll, Heinz-Georg Jahnke, Andrea A. Robitzki, Roman Metzger, Marco Metzger

**Affiliations:** ^1^CELLT Research Group, Translational Centre for Regenerative Medicine, University of LeipzigLeipzig, Germany; ^2^Department of General and Visceral Surgery, St. George's HospitalLeipzig, Germany; ^3^Department of Tissue Engineering and Regenerative Medicine, University Hospital WuerzburgWuerzburg, Germany; ^4^Division of Molecular biological-biochemical Processing Technology, Center for Biotechnology and Biomedicine (BBZ), University of LeipzigLeipzig, Germany; ^5^Department of Pediatric Surgery, University of LeipzigLeipzig, Germany

**Keywords:** laser microdissection, enteric nervous system, myenteric plexus, aging, sodium channels

## Abstract

The enteric nervous system (ENS) poses the intrinsic innervation of the gastrointestinal tract and plays a critical role for all stages of postnatal life. There is increasing scientific and clinical interest in acquired or age-related gastrointestinal dysfunctions that can be manifested in diseases such as gut constipation or fecal incontinence. In this study, we sought to analyze age-dependent changes in the gene expression profile of the human ENS, particularly in the myenteric plexus. Therefore, we used the laser microdissection technique which has been proven as a feasible tool to analyze distinct cell populations within heterogeneously composed tissues. Full biopsy gut samples were prepared from children (4–12 months), middle aged (48–58 years) and aged donors (70–95 years). Cryosections were histologically stained with H&E, the ganglia of the myenteric plexus identified and RNA isolated using laser microdissection technique. Quantitative PCR was performed for selected neural genes, neurotransmitters and receptors. Data were confirmed on protein level using NADPH-diaphorase staining and immunohistochemistry. As result, we demonstrate age-associated alterations in site-specific gene expression pattern of the ENS. Thus, in the adult and aged distal parts of the colon a marked decrease in relative gene expression of neural key genes like *NGFR*, *RET*, *NOS1* and a concurrent increase of CHAT were observed. Further, we detected notable regional differences of RET, CHAT, TH, and S100B comparing gene expression in aged proximal and distal colon. Interestingly, markers indicating cellular senescence or oxidative stress (*SNCA*, *CASP3*, *CAT*, *SOD2*, and *TERT*) were largely unchanged within the ENS. For the first time, our study also describes the age-dependent expression pattern of all major sodium channels within the ENS. Our results are in line with previous studies showing spatio-temporal differences within the mammalian ENS.

## Introduction

The gut is comprised of three distinct tissue regions i.e., the mucosa, submucosa, and muscle layers, which are innervated by the diverse network of the enteric nervous system (ENS). This neuronal network is composed of two main plexuses: the myenteric plexus (Auerbach's) is located between the outer longitudinal and inner circular muscle layer and is responsible for smooth muscle function, and the submucosal plexus (Meissner's), which is situated between inner circular muscle and mucosa, mainly regulating secretion processes and blood flow. Glial cells provide structural supplement to the neuronal network. They also perform metabolic, trophic and protective functions (Gershon and Rothman, 1991; Cabarrocas et al., 2003; Ruhl et al., 2004; Nasser et al., 2006).

Decreasing birth rates and improved health care have both contributed to our aging population. Typical age-related diseases such as gut constipation can be correlated with alterations and decreased regenerative potential in the ENS (Saffrey, 2013). This is emphasized by manifestation of diseases such as slow-transit constipation, chronic intestinal pseudo-obstruction, achalasia, or inflammatory enteric neuropathies (Talley et al., 1992; Majumdar et al., 1997; Ratnaike, 1999; De Lillo and Rose, 2000). Despite this, relatively little is known about the regulatory mechanisms and specific molecular pathways involved. Nonetheless, studies of the gut in both humans and rodents have generally revealed an age-related reduction in the number of enteric neurons, mainly within the myenteric plexus. Age-related damage to specific neuronal populations has been well characterized in rodents (El-Salhy et al., 1999; Phillips and Powley, 2001; Phillips et al., 2003, 2004; Wade and Cowen, 2004). Furthermore, the colon of aged rodents has been shown to be functionally changed with a significant reduction in defecation frequency and stool quantity (McDougal et al., 1984; Smits and Lefebvre, 1996). Furthermore, a loss up to 60% of number of neurons has been demonstrated in the distal colon of guinea pigs (Gabella, 1989) and rats (Santer and Baker, 1988). Such experimental findings have been functionally correlated and compared to results for humans. Morphologically alterations while aging in humans are proven by an onset of decline of enteric neuronal cells in early childhood, at the age of four (Wester et al., 1998, 1999) and increased to a loss of 37% at the age up to 65 years (Gomes et al., 1997).

The normal occurring plasticity of the ENS characterized by changes of the shape of the ganglia due to peristalsis was already described in 1990 (Gabella, 1990). Ongoing studies dealt with the changes in gross morphology of myenteric plexus during aging (Saffrey, 2013). In 2004 a study was published showing the morphological alterations of ganglia during aging such as hypertrophy (Hanani et al., 2004). Hanani et al. (2004) categorized myenteric ganglia into three types: normal, those containing empty spaces (“cavities”), and those containing large nerve fiber bundles. They reported of an enlargement of the ganglia due to arising cavities and postulated that a decrease in the proportion of normal ganglia occurs with increasing age. Indeed, deficits in distinct neuronal subpopulations such as intrinsic primary afferent neurons (IPANS, Feher and Penzes, 1987; Van et al., 2001) or cholinergic neurons (Wade, 2002; Bernard et al., 2009) have been observed in rodent and human tissue.

Neural signal transmission in ENS is controlled by voltage-gated channels and receptor mediated signal transduction. The voltage-gated channels may be specific for potassium, calcium, or sodium and comprise one alpha and two beta subunits (Beneski and Catterall, 1980; Hartshorne and Catterall, 1981; Hartshorne et al., 1982). Alterations in voltage-gated channels provide potent pharmacological targets for the treatment of a variety of functional disturbances of the gut including age-related diseases. Potential targets of interest include serotoninergic, muscarinic, dopaminergic or motilin receptors (De and Camilleri, 2004). Furthermore, it has also been hypothesized that age-related alterations in neurotransmission may be a result of particular loss or adjustment in the expression of sodium channels within the enteric plexus. Sodium channels can be distinguished in different isoforms such as Na_v_1.1 to Na_v_1.9 or to their reaction of nanomolar concentrations of the sodium blocker tetrodotoxin. Previous studies demonstrated their presence and function in the central and peripheral nervous system (PNS) including the ENS (summarized in Table 1).

**Table 1:** **Characterization of sodium channels Na_v_1.1-1.9**.

**Na_n_ (Gene symbol)**	**TTX-sensitivity**	**CNS**	**PNS**	**ENS nerval plexus**	**Muscle**	**References**
Na_v_1.1 (*SCN1A*)	+	++	+	−	−	Noda et al., 1986; Felts et al., 1997; Strege et al., 2003; Bartoo et al., 2005; Sage et al., 2007
Na_v_1.2 (*SCN2A*)	+	++	+	+	n.d.	Noda et al., 1986; Felts et al., 1997; Holm et al., 2002; Hanani et al., 2004; Bartoo et al., 2005
Na_v_1.3 (*SCN3A*)	+	++	+	+	n.d.	Kayano et al., 1988; Beckh, 1990; Felts et al., 1997; Hanani et al., 2004; Bartoo et al., 2005; Sage et al., 2007
Na_v_1.4 (*SCN4A*)	+	+	+	+	+	Deshpande et al., 2002; Ou et al., 2002; Hanani et al., 2004; Bartoo et al., 2005; Candenas et al., 2006; Sage et al., 2007
Na_v_1.5 (*SCN5A*)	−	+	+	+	+	Deshpande et al., 2002; Holm et al., 2002; Ou et al., 2002; Strege et al., 2003; Hanani et al., 2004; Bartoo et al., 2005; Candenas et al., 2006; Sage et al., 2007
Na_v_1.6 (*SCN8A*)	+	++	+	+	n.d.	Schaller et al., 1995; Felts et al., 1997; Raman et al., 1997; Krzemien et al., 2000; Tzoumaka et al., 2000; Bartoo et al., 2005
Na_v_1.7 (*SCN9A*)	+	+	++	+	n.d.	Hanani et al., 2004; Bartoo et al., 2005; Candenas et al., 2006; Sage et al., 2007
Na_v_1.8 (*SCN10A*)	−	−	+	−	−	Ou et al., 2002; Rugiero et al., 2003; Hanani et al., 2004; Bartoo et al., 2005; Sage et al., 2007
Na_v_1.9 (*SCN11A*)	+	+	+	+	+	Ou et al., 2002; Rugiero et al., 2003; Hanani et al., 2004; Bartoo et al., 2005; Candenas et al., 2006; Sage et al., 2007; Copel et al., 2013

*DRG, dorsal root ganglia; n.d., not determined*.

Our study aimed to confirm age-dependent gene expression changes of ENS, particularly in ganglia of myenteric plexus using laser microdissection. Due to their biological significance within the ENS we also included analysis of sodium channels. Taken the comparative data there is evidence for decreased gene expression going along with loss of neural cells itself.

## Materials and methods

### Tissue

**Figure 1:**
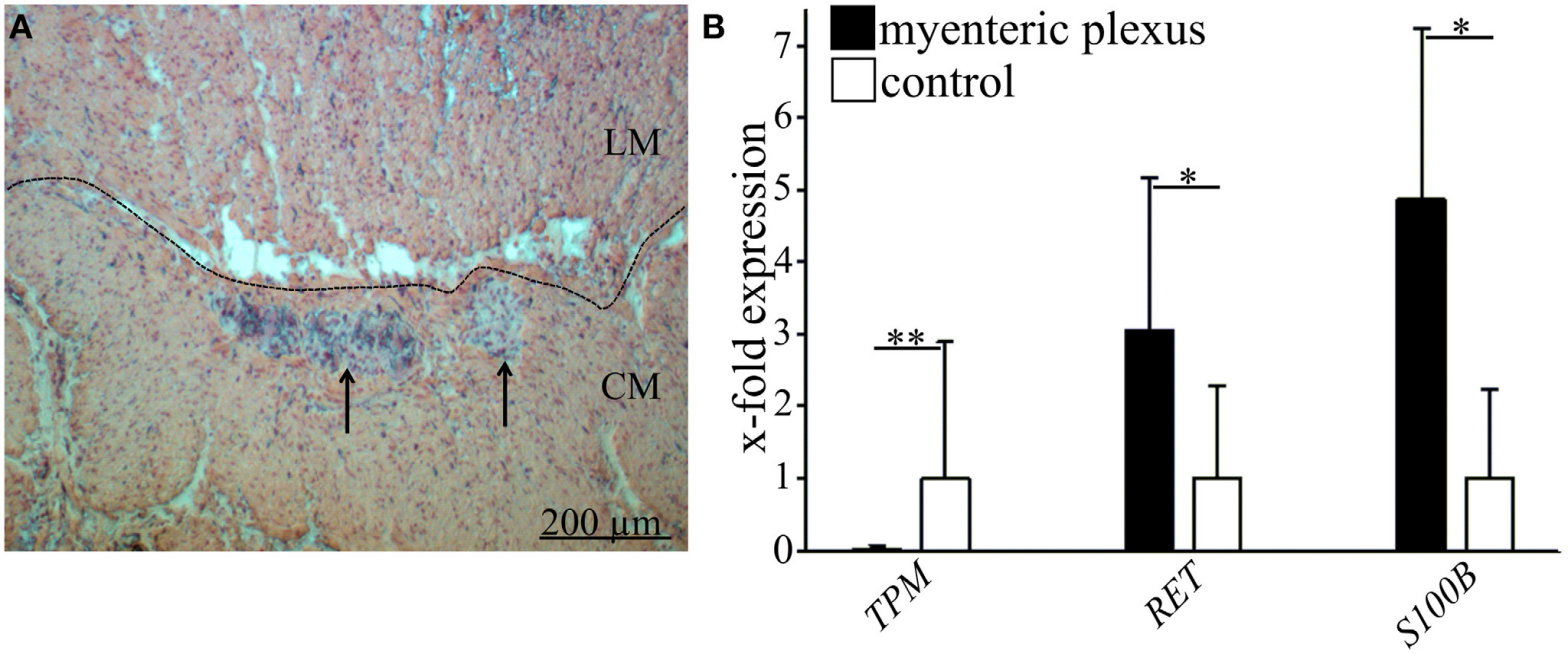
**Identification of enteric ganglia of human myenteric plexus and validation of laser microdissection technique. (A)** Histological staining of large intestine with hematoxylin and eosin. Ganglia (marked by arrows) are visible between the outer longitudinal (LM) and the inner circular muscle (CM). Scale bar 200 μm. **(B)** Representative validation of laser microdissection technique by qRT-PCR in one donor. Black bars indicate the relative gene expression of tropomyosin (*TPM1*, smooth muscle marker, ^**^P ≤ 0.005), Ret receptor (*RET*, neuronal marker, ^*^P ≤ 0.05) and S100 calcium binding protein B (*S100B*, glial marker, ^*^P ≤ 0.05) compared to full wall tissue controls (white bars). Data demonstrate that TPM is almost absent in microdissected myenteric plexus whereas Ret and S100 beta are both significantly enriched.

All experiments were performed with human colonic tissue, which was obtained under ethical approval and with fully informed consent (Department of General and Visceral Surgery, St. George's Hospital of Leipzig, Germany and Department of Pediatric Surgery, University of Leipzig, Germany). After inspection by pathology “healthy” full-thickness gut samples were obtained from donors undergoing gut resection surgery (see Table 2 for diagnoses). After surgery the tissue was immediately stored in Ca^2+^- and Mg^2+^-free Hanks PBS (PAA, Coelbe, Germany) and embedded in OCT compound media (Weckert Labortechnik, Kitzingen, Germany). The samples were stored at −80°C before being cryosectioned and stained for hematoxylin and eosin (H&E) or by immunofluorescence. Based on morphological abnormalities shown by H&E-staining all samples were analyzed regarding the presence of myenteric ganglia. Structures of myenteric plexus (arrows in Figure 1A) were distinguished from surrounding longitudinal (LM) and circular muscle (CM) (Figure 1A). Samples of four children (3 M, 1 F; 4–12 months), four adult (1 M, 3 F; 48–58 years) and eleven aged donors (4 M, 7 F; 70–95 years) were included into the study and analyzed by comparative quantitative Polymerase Chain Reaction (qPCR) after structural analysis using a Nikon intenselight C-HGFI microscope (Nikon, Melville, USA) (Table 2).

**Table 2:** **Human tissue samples used in the study**.

**Donor age**	**Gender**	**Tissue**	**Pathology**
4 months	Male	Distal colon	Anal atresia
7 months	Male	Distal colon	Anal atresia
10 months	Male	Distal colon	Anal atresia
1 year	Female	Distal colon	Anus praeter
48 years	Female	Sigma	Cancer
52 years	Female	Sigma	Cancer
55 years	Female	Colon descendens	Polyps
58 years	Male	Sigma	Cancer
70 years	Male	Colon ascendens	Cancer
72 years	Male	Colon descendens	Cancer
74 years	Female	Colon transversum	Cancer
77 years	Male	Sigma	Diverticulitis
78 years	Female	Sigma	Diverticulitis
82 years	Female	Sigma	Cancer
84 years	Male	Colon transversum	Cancer
85 years	Female	Sigma	Cancer
85 years	Female	Sigma	Cancer
91 years	Female	Colon ascendens	Cancer
95 years	Female	Colon transversum	Cancer

### Laser microdissection

In order to isolate human myenteric plexus from human gut tissue laser microdissection was used as described previously (Bottner et al., 2010). PEN-membrane coated slides (Carl Zeiss, Jena, Germany) were rinsed with *RNAseZAP™* (Sigma) and irradiated with UV-light (920 J/cm^2^, HL-2000 HybrilinkerTM with UV-transillumination, VWR, Darmstadt) to eliminate RNases and create a more hydrophobic surface. The tissue was cryosectioned in 16 μm slices and stained with hematoxylin and eosin (H&E). Dried slides were placed under an inverted microscope (Nikon Instruments) with the *NanoElectricBeam-Laser device* (Stuhrmann et al., 2006) and dissected under 20× magnification. For isolation the desired tissue was marked within the life-view screen of the controlling software (TWUT 2010) followed by automatic cutting using a highly focused and precise guided UV laser-beam (337 nm, 20 μJ/pulse). Dissection of the PEN-membrane enabled sharp edged and high selectively defined types of tissue, such as muscle or ganglia, into the lid of a collecting tube. The lid was lined with RNAlater® (Qiagen, Hilden, Germany) to stabilize the RNA. A total of 50 ganglia and 50 comparable sized pieces of muscle (circular and longitudinal muscle layer) were collected from each donor. Samples were then pooled for each tissue type and each individual donor. Total RNA was isolated using *RNeasy® Plus Micro Kit* (Qiagen). Maximal amounts of RNA were encoded by reverse transcriptase (Platinum® Taq DNA Polymerase, Invitrogen, Karlsruhe, Germany) and gene expression analysis conducted.

### Quantitative polymerase chain reaction (qPCR)

According to manufacturer's protocol the *QuantiTect*® *SYBR*® *green PCR Kit* (Qiagen) was performed on ABI 7500 (ABI, Darmstadt, Germany) for 42 cycles (C_(T)_). The primers (Table 3, Invitrogen) were designed for amplifying the housekeeping genes glyceraldehyde-3-phosphate dehydrogenase (*GAPDH*), hypoxanthine phosphoribosyltransferase 1 (*HPRT1*) and mitochondrial ribosomal protein L15 (*MRPL15*). Primers against pan-neural genes representatively shown by nerve growth factor receptor (neurotrophin receptor p75, *NGFR*) and pan neuronal markers like ret proto-oncogene or Ret receptor (*RET*) were designed in addition to primers targeting the genes for the neuronal subtypes choline acetyltransferase (*CHAT*), nitric oxide synthase 1 (*NOS1*), and tyrosine hydroxylase (*TH*). Other primers detected S100 calcium binding protein B (*S100B*) or tropomyosin 1 (alpha) (*TPM1*) to ensure the quantification of glial and muscle gene expression. To visualize processes of senescence in the myenteric plexus of aged donors gene expression of α-synuclein (*SNCA*), caspase-3 (*CASP3*), catalase (*CAT*), superoxide dismutase 2 (*SOD2*), and telomerase reverse transcriptase (*TERT*) were analyzed. Further, the whole expression pattern of all major sodium channels was investigated by gene expression analysis of Na_v_1.1 (*SCN1A*), Na_v_1.2 (*SCN2A*), Na_v_1.3 (*SCN3A*), Na_v_1.4 (*SCN4A*), Na_v_1.5 (*SCN5A*), Na_v_1.6 (*SCN8A*), Na_v_1.7 (*SCN9A*), Na_v_1.8 (*SCN10A*), and Na_v_1.9 (*SCN11A*). By analysis of melting curves and agarose gel electrophoresis the quality of amplified PCR products was verified. Data of qPCR study were analyzed by the 2(-Delta Delta C(T)) method and normalized to mean of three different housekeeping genes. The gene expression panels of the aged donors were then comparatively visualized to the average gene expression of the young donors.

**Table 3:** **Oligonucleotides used in the study**.

**Gene (Gene symbol)**	**Primer 5′ → 3′ (sense, antisense)**	**Genbank no**.	**T_An_ (°C)**	**Product-length (bp)**	**References**
Glyceraldehyde-3-phosphate dehydrogenase (*GAPDH*)	accacagtccatgccatcac	NM_002046	59	452	Metzger et al., 2007
	tccaccaccctgttgctgta				
Hypoxanthine phosphoribosyl-transferase 1 (*HPRT*)	tgaacgtcttgctcgagatgtg	NM_000194.2	60	125	Bottner et al., 2010
	ccagcaggtcagcaaagaattt				
Mitochondrial ribosomal protein L15 (*MRPL15*)	Unknown	NM_014175	60	125	QuantiTect Primer Assay 200; Qiagen
Neurotrophin receptor p75, (*NGFR*)	tgagtgctgcaaagcctgcaa	NM_002507	60	230	Tsunoda et al., 2006
	tctcatcctggtagtagccgt				
Ret receptor (*RET*)	agatttcggatttcggcttgt	NM_020630.4	63	161	Metzger et al., 2009
	ccacagcaggacaccaaaaga	NM_020975.4			
		NM_020630.4			
		NM_020975.4			
Cholinaceyl transferase (*CHAT*)	tggtgcaatcagttctttgt	NM_020549.3	55	239	Designed by using NCBI/Primer-BLAST
	aggcagatgcagcgctcaatcatgtc	NM_020984.2			
		NM_020986.2			
		NM_020985.2			
Nitric oxide synthase (*NOS1*)	tccctcctcgggcttctcgc	NM_000620.2	60	193	Designed by using NCBI/Primer-BLAST
	cccacagcgacggccatgtt				
Tyrosine hydroxylase (*TH*)	acggtggagttcgggctgtg	NM_199293.2	60	248	Designed by using NCBI/Primer-BLAST
	aaggggcgctggatgcgtg	NM_199292.2			
		NM_000360.3			
S100 calcium binding protein B (*S100B*)	aggacccgcagcagagacga	NM_006272.2	60	102	Designed by using NCBI/Primer-BLAST
	tcgatgagggccaccatggc				
Tropomyosin 1 (alpha) (TPM)	ctcgcagaaggaagacagatatgag	NM_001018020	60	101	Bottner et al., 2010
	tagttactgacctctccgcaaactc				
α-Synuclein (*SNCA*)	tcacgccttgccttcaagccttct	NM_000345.3	60	148	Designed by using NCBI/Primer-BLAST
	ccacaactccctccttggcctt				
Caspase 3 (*CASP3*)	cctgctcacactcggcgctc	NM_004346.3	60	172	Designed by using NCBI/Primer-BLAST
	tccagagtccattgattcgcttcca				
Catalase (*CAT*)	gcccgatgtgcatgcaggaca	NM_001752.3	60	167	Designed by using NCBI/Primer-BLAST
	tgcccgcacctgagtaacgt				
Superoxide dismutase 2 (*SOD2*)	aggctcaggttggggttggct	NM_000636.2	60	135	Designed by using NCBI/Primer-BLAST
	gcgtgctcccacacatcaatccc				
Telomerase reverse transcriptase (*TERT*)	gctgctcaggtctttcttttatgtc	NM_198253.2	60	116	Designed by using NCBI/Primer-BLAST
	tcaagtgctgtctgattccaatg				
Na_v_1.1 (*SCN1A*)	gaagaacagcccgtagtggaa	NM_006920.4	60	225	Candenas et al., 2006
	ttcaaatgccagagcacca	NM_006920.4			
Na_v_1.2 (*SCN2A*)	gaaggcaaagggaaactctgg	NM_001040143.1	60	297	Candenas et al., 2006
	cagtgagacatcaacaatcaggaag	NM_001040142.1			
		NM_021007.2			
Na_v_1.3 (*SCN3A*)	aaaccccaactatggctacacaa	NM_001081677.1	60	367	Candenas et al., 2006
	tcctaacccacctattccactga	NM_006922.3			
		NM_001081676.1			
Na_v_1.4 (*SCN4A*)	caacaacccctacctgaccatac	NM_000334.4	60	317	Candenas et al., 2006
	gcagagtccaccacttcttcc				
Na_v_1.5 (*SCN5A*)	ccgccatttacacctttgagt	NM_001099405.1	60	294	Candenas et al., 2006
	cgctgaggcagaagactgtg	NM_001099404.1			
		NM_000335.4			
		NM_198056.2			
Na_v_1.6 (*SCN6A*)	aaggttgtgtccagcggttc	NM_014191.2	60	207	Candenas et al., 2006
	ggatggtgcggatggtctt				
Na_v_1.7 (*SCN9A*)	cccacagaccccaggagcga	NM_002977.2	60	180	Designed by using NCBI/Primer-BLAST
	tggtcgtgccctctggcaga				
Na_v_1.8 (*SCN10A*)	tggaattccccattggatccctcg	NM_006514.2	60	175	Designed by using NCBI/Primer-BLAST
	gctttcaagtccagctggggcc				
Na_v_1.9 (*SCN11A*)	ggcaggctgttttattcccgcc	NM_014139.2	60	151	Designed by using NCBI/Primer-BLAST
	tgcagccagagagtcggaagtga				

### Immunohistochemistry

Cryosectioned OCT-compound-embedded tissue was fixed with 4% phosphate-buffered paraformaldehyde (PFA, Sigma, Steinheim, Germany) for 10 min at room temperature (RT) and rinsed three times in phosphate buffered saline (PBS, PAA). After 30 min blocking at RT (PBS containing 0.3% Triton X-100 and 10% donkey serum, PAA) sections were treated with following primary antibodies diluted in PBS containing 0.1% Triton X-100 (Sigma) overnight at 4°C: rabbit anti-neurotrophin receptor p75 (p75, 1:250; Promega, Mannheim, Germany), rabbit anti-S100 beta (S100 beta, 1:600, Dako, Glostrup, Denmark), mouse anti-Na_v_1.6 (1:200, Santa Cruz Biotechnology, Heidelberg, Germany) and goat anti-Na_v_1.9 (1:200, Santa Cruz Biotechnology). After washing with PBS three times, the sections were incubated with fluorochrome-conjugated secondary antibodies: donkey anti-rabbit Alexa488 (1:400, Dianova, Hamburg, Germany), donkey anti-mouse Alexa488 (1:400, Dianova) and donkey anti-goat Cy3 (1:400, Dianova) for 30 min at room temperature. After three washes in PBS sections were additionally stained with DAPI (4′-6-Diamidino-2-phenylindole) solution (1 μg/mL, Roth, Karlsruhe, Germany) in PBS for 10 min. Stained sections were rinsed in PBS and cover slipped with Kaiser's gelatin (Merck, Darmstadt, Germany). Image capturing and analysis was done on Nikon eclipse Ti inverted microscope. All figures were assembled and annotated using Corel Draw X4 (v14.0.0.567 software; Unterschleissheim, Germany) and ImageJ (v1.38; National Institutes of Health, Bethesda, USA).

### Whole-mount NADPH-diaphorase staining

Gut tissue was cryo-embedded, sliced and fixed with 4% PFA overnight. Tissue was then incubated in a solution of 3 ml PBS, 1.5 mg nitroblue tetrazozolium (Sigma), 3 mg β-*Nicotinamide adenine dinucleotide phosphate* (β-NADPH, Sigma) and 1.5 μl Triton X-100 (Sigma) at 37°C for 30 min in the dark (Wallace et al., 2009). Sections were then washed in PBS and nuclear fast red staining (Vector Laboratories Inc., Burlingame, USA) was performed by incubation with the coloring agent for 20 min at room temperature. The reaction was stopped by washing in PBS. The NADPH-staining was preserved by covering the tissue sections with Kaiser's gelatine (Merck) and cover slips. Samples were visualized and analyzed under morphological peculiarities with Nikon intenselight C-HGFI (Nikon). For quantification, NOS-positive cells in the plexus myentericus region were counted microscopically (25× objective) from 5 non-consecutive longitudinal serial cryosections (16 μm × ~1.5 cm) of each donor (children *n* = 4, aged donors *n* = 6).

### Whole-mount senescence associated β-Galactosidase Staining

In order to verify increased senescence in aged human ENS *Senescence* β- *Galactosidase Staining Kit* (Cell Signaling, Frankfurt, Germany) was used according to the manufacturer's instructions. Human cryosectioned colonic tissue was fixed for 10 min and then incubated with the coloring agent over night at 37°C. The staining was stopped by washing the slides in PBS, slides were then preserved by covering sections with gelatin-rinsed cover slips. Slides were analyzed under NIKON *eclipse* TS 100 (Nikon).

### Statistical analysis

All data were obtained from at least three independent biological samples and partly visualized as box-and-whisker plots with median and data range (minimum and maximum) or column charts. Statistical analysis was analyzed by analysis of variance (ANOVA) using Sigma Plot for Windows Version 11. A *p*-value of ≤ 0.05 was considered significant, a *p*-value between 0.05 and 0.1 was considered as moderate evidence for statistical significance with biological relevance.

## Results

### Alterations of neural gene expression in ganglia of adult and aged donors

Firstly, to validate the specificity of the used method of laser microdissection, the tissue-specific gene expression of *TPM1* (smooth muscle-like cells), *RET*, and *S100B* (neurons and glia) was demonstrated for lasermicrodissected regions of plexus myentericus and whole tissue slices as control (Figures 1A,B).

**Figure 2:**
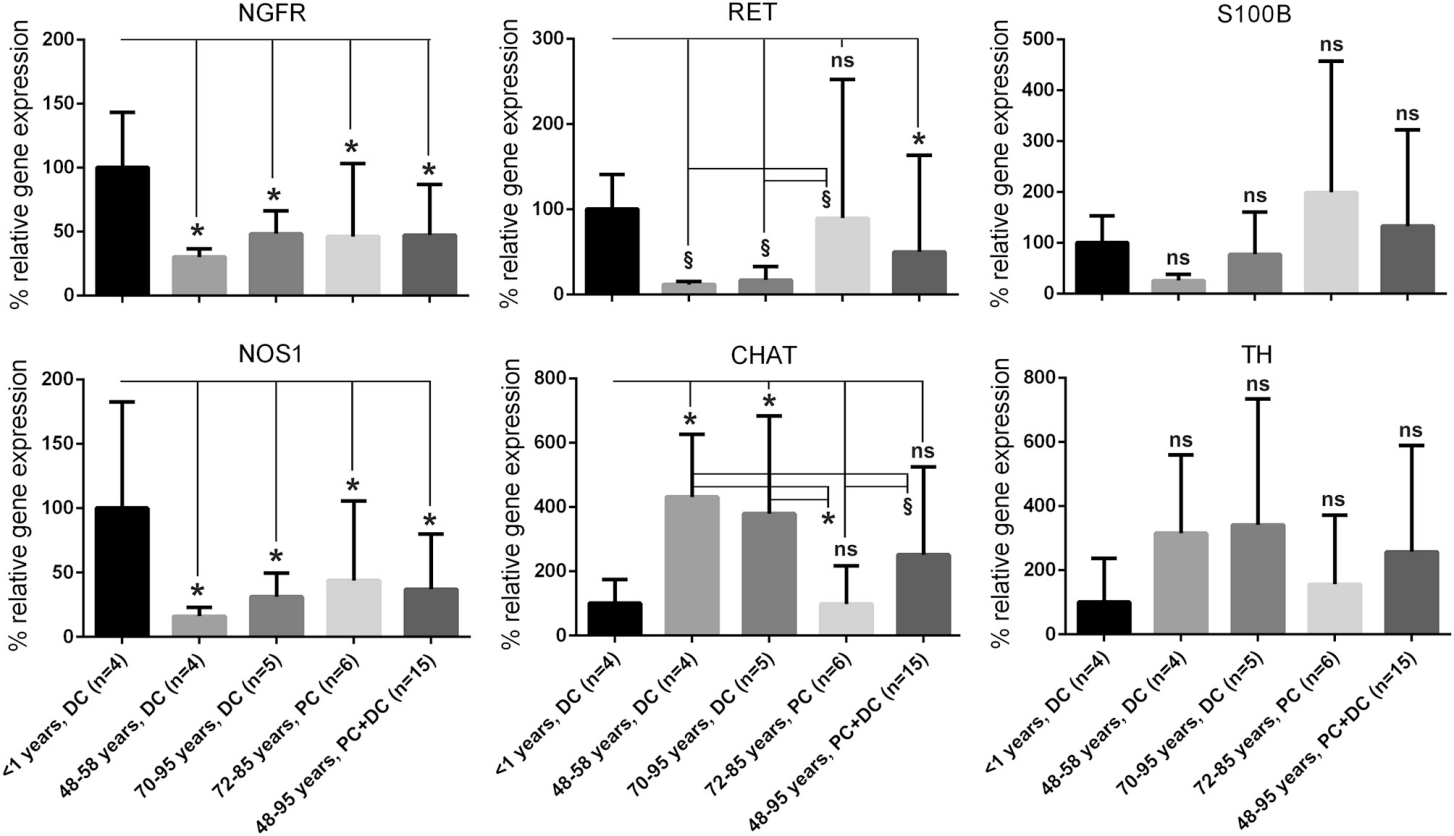
**Quantification of gene and protein expression of markers characteristic for ENS**. Analysis of a panel of markers characteristic for ENS confirms that the plexus from aged donors show significant decrease of neurotrophin receptor p75 and Ret receptor (*NGFR* and *RET*, identifying ENS plexus) and nitric oxide synthase (*NOS1*, labeling a main neuronal subpopulation), whereas other neural subtypes were up-regulated like choline acetyltransferase (*CHAT*). Other genes such as tyrosine hydroxylase (*TH*) or the glial marker S100 calcium binding protein B (*S100B*) were not significantly regulated. These data suggest general alterations of ENS integrity during aging. Depending on gut age and region, different subpopulations seem to be differentially affected. ^*^P ≤ 0.05 and 0.05 <^§^*P* < 0.1. DC, distal colon; PC, proximal colon; ns, not significant.

**Figure 3:**
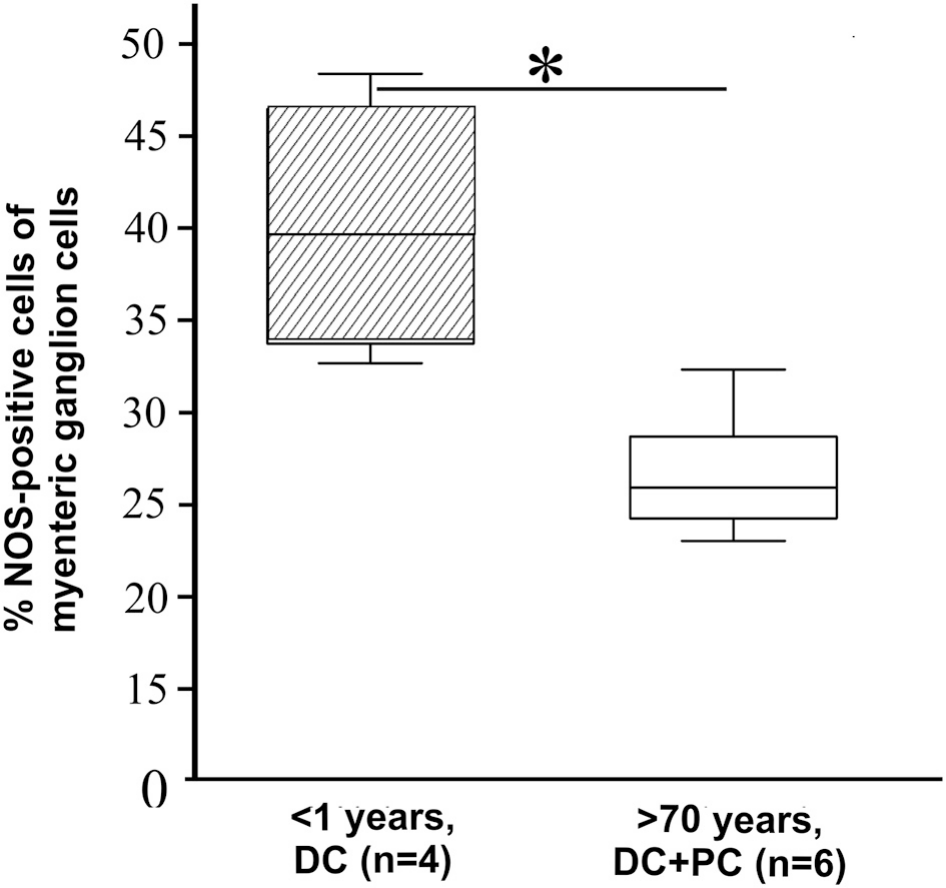
**Number of NOS-positive neurons**. Examination of nitric oxide synthase- (NOS) positive cells by using NADPH-diaphorase staining compared to all plexus cells counted by nuclear fast red. Similar to the significantly reduced mRNA gene expression the number of NOS-positive neurons showed a significant decline (37.3 ± 9.1 to 20.7 ± 4.2%) in aged donors and verified the alteration of gene expression on protein level. ^*^P ≤ 0.05.

**Figure 4:**
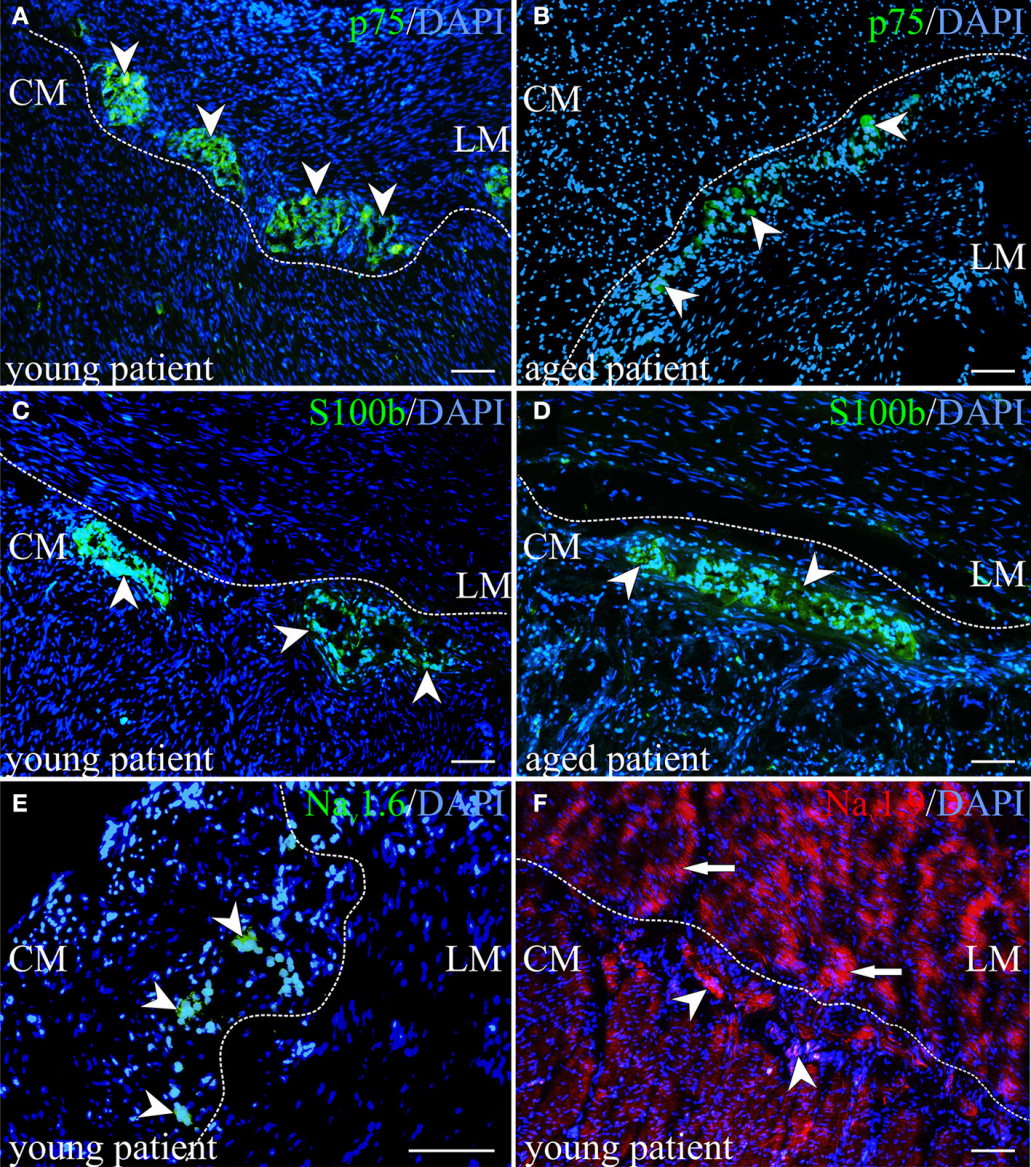
**Immunohistochemical visualization of neurotrophin receptor p75 (p75), S100 calcium binding protein B (S100 beta) and sodium channels. (A,B)** Detection of the protein for neurotrophin receptor p75 underpinned assumptions of less number of neural progenitor cells in aged human myenteric plexus. The number of p75-positive cells marked by arrowheads could be shown in higher number in young tissue in **(A)** compared to aged colon in **(B)**. **(C,D)** In contrast, a slight relative increase of S100 beta-expressing cells was observed in aged tissues of proximal colon. **(E)** Although slight cross-reaction with cell nuclei in the muscle tissue was detected, sodium channel Na_v_1.6 could be demonstrated exclusively in myenteric plexus, representatively shown for one child and marked by arrowheads. **(F)** The sodium channel subtype Na_v_1.9 was identified in ganglion cells (marked by arrowheads) and in muscle tissue (marked by arrows). **(A–F)** outer longitudinal (LM), inner circular muscle (CM); nuclei stained with DAPI (blue); scale bar 100 μm.

Afterwards, a selected gene expression panel was analyzed in the microdissected ENS of distal colon from babies (<1 year), adults (48–58 years) and aged donors (70–95 years) showing that the pan-neural marker *NGFR* (3.3 and 2.1-fold) and the pan-neuronal marker *RET* (8.5 and 6.0-fold) were significantly decreased during aging, respectively (adults and aged donors vs. babies, respectively; Figure 2). The same was true for nitric oxide synthase 1 (*NOS1*), which catalyzes the generation of nitric oxide, the most important non-cholinergic non-adrenergic inhibitory neurotransmitter. A significant 6.2 and 4.3-fold decline of gene expression for the neuronal subtype marker *NOS1* was detected, whereas the gene expression for choline acetyltransferase (*CHAT*) significantly increased by 4.3 and 3.8-fold, respectively. Due to a relatively high variability between tissues we could not detect statistically significant effects on tyrosine hydroxylase (TH) or S100B gene expression. However, we noticed a mean increase of TH by 3.1 and 3.8-fold and S100B by 3.9 and 2.0-fold. Interestingly, by comparing the more proximal to the distal parts of the colon we were able to detect differences in RET, CHAT, TH, and S100B expression suggesting also regional differences within the ENS. Finally, we qualitatively confirmed our qPCR data by representative analysis on protein level. The change in gene expression can indeed be correlated with an altered cell number proven by the staining for NADPH-diaphorase. It visualized the NOS-positive cells and revealed their loss in the plexus myentericus of the aged donors compared to the babies (Figure 3). In Figure 4 we visualized representative p75- and S100B-expressing cells of young and old donors (arrowheads in Figures 4A–D). Due to a broad staining pattern counting of these cells is not feasible on histological sections.

### No significant increase of senescence markers in samples of aged donors

To evaluate molecular signaling of senescence in colonic tissue we examined gene expression patterns targeting *SNCA*, *CASP3*, *CAT*, *SOD2*, and *TERT*. However, no differences in the expressions of these genes could be demonstrated in the myenteric plexus, the muscle or in the overall tissue of neither young nor aged donors. Senescence was also investigated at the protein level by performing a β-galactosidase staining, however no significant staining could be detected in either the young or aged plexus (data not shown).

### Age-related sodium channel subtype expression in the ENS

For the first time, the complete identification of all major sodium channels was shown in laser microdissected ENS. An overview of our findings in colonic myenteric plexus and circular/longitudinal muscle is shown in Table 4. Thus, gene expression for sodium channels *SCN1A*, *SCN2A*, *SCN3A*, *SCN5A*, *SCN6A*, *SCN9A*, and *SCN11A* could be evidently shown in myenteric plexus of human colon in the majority of analyzed samples. Due to the high number of tissues correlated with the gene expression of *SCN2A*, *SCN3A*, *SCN9A*, and *SCN11A*, these sodium channel subtypes may have significant functional contributions to the excitability of specific populations of enteric neurons and alteration of them could be important in disease.

**Table 4:** **Results for gene expression of sodium channels**.

**Na_n_**	**Result for colonic tissue in current study**	**Myenteric plexus**	**Colonic muscle**
Na_v_1.1	Expression exclusive in plexus	Children 4/4 Aged donors 3/6	Children 0/4 Aged donors 0/6
Na_v_1.2	Expression in plexus and in muscle tissue	Children 3/4 Aged donors 5/6	Children 2/4 Aged donors 2/6
Na_v_1.3	Expression in plexus and in muscle tissue (one child only)	Children 4/4 Aged donors 5/6	Children 1/4 Aged donors 0/6
Na_v_1.4	Expression exclusive in muscle tissue of three children	Children 0/4 Aged donors 0/6	Children 3/4 Aged donors 0/6
Na_v_1.5	Expression exclusive in plexus	Children 4/4 Aged donors 3/6	Children 0/4 Aged donors 0/6
Na_v_1.6	Expression exclusive in plexus	Children 3/4 Aged donors 2/6	Children 0/4 Aged donors 0/6
Na_v_1.7	Expression in plexus and in muscle tissue	Children 4/4 Aged donors 5/6	Children 4/4 Aged donors 3/6
Na_v_1.8	Expression in plexus (one aged donor only)	Children 0/4 Aged donors 1/6	Children 0/4 Aged donors 0/6
Na_v_1.9	Expression in plexus and in muscle tissue (two children)	Children 4/4 Aged donors 4/6	Children 2/4 Aged donors 0/6

The gene expression of *SCN4A* was not detected at all in the plexus and *SCN10A* only in one of the aged specimen indicating that these two channels might play a minor role in the ENS. Interestingly, gene expression of sodium channels *SCN1A*, *SCN5A*, *SCN6A*, and *SCN10A* could be found exclusively in myenteric plexus and not muscle tissue. Noteworthy, only gene expression of channel *SCN9A* was detected in most of the analyzed muscle samples, in contrast to the remaining channels (*SCN2A*, *SCN3A*, *SCN4A*, *SCN11A*), which were present only in a few donors.

With respect to age-dependent alterations, we did not observe an “all or nothing” effect, however genes for the channels *SCN1A*, *SCN3A*, *SCN5A*, *SCN9A*, and SCN11A were expressed in the ENS of all children but only in part in the elderly. In the muscle, the same effect was true only for channel *SCN9A*. Overall, with our qPCR analysis we were able to identify channels, which are only expressed in the ENS and two of those, *SCN1A* and *SCN5A*, seem to be additionally altered during aging. For two representative channels we qualitatively confirmed our data on protein level via immunohistochemistry (Figures 4E–F). Thus, channel Na_V_1.6 represents a candidate exclusively expressed in the ENS and Na_v_1.9 one expressed in both, the ENS and muscle tissue.

## Discussion

Our study has confirmed that age-associated alterations in site-specific gene expression patterns occur within the ENS. We have demonstrated an obvious decrease in the neural gene expression for *NGFR*, in addition to the neuronal genes *RET* and *NOS1* as well as an increase in gene expression for the cholinergic marker *CHAT* in tissue samples of myenteric plexus in distal large intestine of aged donors compared to children. Furthermore, we described the gene expression patterns for sodium channels within colonic tissue by distinguishing clearly between myenteric plexus and colonic muscle tissue.

Morphological alterations within the ENS in early childhood are well known (Wester et al., 1999) and a decline of 37% of neurons in aged donors older than 65 years compared to donors aged between 20 and 35 was demonstrated (Gomes et al., 1997). However, this phenomenon in the aging human intestines remains poorly understood. The general deficit of enteric neurons has already been reported in aged guinea pigs (Gabella, 1989). In rats, Santander and Baker confirmed age-related loss of neuronal cells of ENS across all neuronal subtypes and all morphologies and sizes (Santer and Baker, 1988). This was supported by a study in mice done by El-Salhy et al. (1999). Furthermore, they reported of no additional decrease in 24 month old animals, concluding that cell death is completed within the ENS of middle aged rodents. Interestingly, although rodents and humans clearly differ in their postnatal development and physiology, our data suggest a similar phenomenon since we do not see any differences in gene expression between the middle aged and aged groups. In contrast, we noticed differences between tissue regions, which can be expected due to different physioanatomical conditions.

After powerful improvement of histological and molecular biological methods more individual cell types and distinct populations could previously be examined and functional classes of enteric neurons could be distinguished by their molecular signatures (Furness, 2000). Degeneration within distinct enteric neuronal subpopulations due to aging has now been demonstrated by a number of studies (Wu et al., 2003; Thrasivoulou et al., 2006). In 2004, Phillips et al. showed a general decline in the number of neural cells within the ENS of aged rodents (Phillips et al., 2004). This was associated with a decline in substance P-, somatostatin- and vasoactive intestinal peptide (VIP)-positive fibers in rats (Phillips and Powley, 2001). Indeed, our results for decline in gene expression patterns of *NGFR*, *RET*, and *NOS1* could underpin the assumption of loss of neuronal cells. To demonstrate the potential relationship between decreased mRNA gene expression and reduced cell number itself we visualized characteristic cells immunostained for neural cell markers like NOS and p75 in young and aged human colonic tissue. Our results are supported by a study of Takahashi and colleagues showing a reduction of more than 50% of NOS-positive neurons in colon of aged rats (Takahashi et al., 2000) underlining assumptions for human constipations while aging (Hays and Roberts, 2006).

Cholinergic neurons are found in a functionally diverse group of neurons, such as excitatory motor neurons that innervate smooth muscle, intrinsic sensory neurons, and interneurons. But if special subpopulations of cholinergic myenteric neurons are selectively vulnerable during aging has not been analyzed so far. Overall, rodent studies gave evidence for significant alterations of choline acetyltransferase-positive cells by showing neurodegeneration in the aged gut (Roberts et al., 1994; Cowen et al., 2000; Phillips et al., 2003), whereas nitrergic neurons mainly seemed to be unaffected (Wu et al., 2003; Thrasivoulou et al., 2006). In 2000 Cowen et al. confirmed a 64% reduction in the number of cholinergic neurons within the ileum of 24-month-old rats, describing a distinct neuronal loss in ENS only in the population of choline acetyltransferase-positive neurons (Wu et al., 2003). In human studies, a significant decrease in choline acetyltransferase-positive neurons within the myenteric plexus of colonic samples of 16 donors (33–99 years old donors, 9 male, 7 female) has been reported by immunohistochemical analyses (Bernard et al., 2009). However, our study demonstrated highly variable gene expression of *CHAT* depending on the tissue region and we rather observed an increase in *CHAT* gene expression. This could be due to the fact that we looked for changes on level of RNA and not of protein and furthermore we analyzed myenteric plexus of children under 1 year and there might be differences in the age-dependent vulnerability of distinct neuronal subtypes of the plexus in the gut.

The age-related expression of glial marker gene *S100B* could be demonstrated for the first time in human ganglia isolated by laser microdissection. Enteric glial cells and their changes during aging have been little studied in rodents before. Thus, with respect to glial cells our results, although not statistically significant, demonstrate the same trend as a previous animal study. In aged Fischer 344 rats (26 month old) compared to their younger counterparts (5–6 months old), S100 beta-positive cells were significantly decreased in the colonic myenteric plexus in all areas except rectum, as determined by immunofluorescence (Phillips et al., 2004).

Furthermore, we should point out, that the analysis and subsequent comparisons of ENS alterations while aging is challenging due to different study designs and a large variation on the proportions of neuronal cells itself depending on the gut region and donor anamnesis. The possible sources of variability in reports of age-related enteric neuronal loss are discussed in detail and reviewed by Saffrey et al. (Saffrey, 2013). Thus, the hypothesis of extensive neuronal loss in the aging gut is not supported by all studies and especially in rodent studies the differences in food intake and the resulting microbial populations play major roles (Cowen et al., 2000). Also, for humans the microbiota is important for the human gastrointestinal health (Aziz et al., 2013) and is known to change while aging (Saffrey, 2013).

In this study, we also reported the distribution of genes for all major sodium channels in young and aged human myenteric plexus in colon in order to aid identification of novel pharmacological targets. Gene expression of sodium channels for *SCN3A*, *SCN4A*, *SCN5A*, *SCN9A*, *SCN10A*, and *SCN11A* which were known to be characteristic for the PNS and rodent ENS (Hanani et al., 2004; Bartoo et al., 2005; Sage et al., 2007) were expected in myenteric plexus of human colon and could be demonstrated except gene expression of *SCN4A* and mostly *SCN10A*.

Furthermore, gene expression for channels commonly found within the central nervous system (CNS) such as *SCN1A*, *SCN3A*, *and SCN6A* were additionally confirmed within the myenteric plexus in the current study. Interestingly, they were almost exclusively expressed within the myenteric plexus and not within the surrounding muscle tissue. Such clear proof of gene expression of *SCN1A* in the ENS, but not in muscle tissue has not previously been reported in rodent studies. Instead, Bartoo et al. demonstrated positive findings for Na_v_1.2, 1.3, 1.6, and 1.7, but not for Na_v_1.1, within the *tunica muscularis* of rodent gut by using RT-PCR and immunohistochemistry (Bartoo et al., 2005).

Na_v_1.5 is known to be expressed in human jejunal muscle and contributes to regulation of intestinal motor function (Holm et al., 2002; Ou et al., 2002; Strege et al., 2003). However, in our study expression in the colon could only be verified in the myenteric plexus of human ganglia (all children and half of aged donors). This result could be due to the restriction to tissue of small intestine in the named studies of Ou et al., Holm et al., and Strege et al. The sodium channels Na_v_1.1 and 1.5 should be pointed out because their gene expression were not only exclusively verified in myenteric plexus, they also showed a 100% confirmation in children and were reduced to 50% in enteric ganglia of aged donors. Our data suggest a decrease in gene expression of these particular channels while aging.

Overall, our data are based on a small number of donors allowing only limited statistics and conclusions. Also we were not able to include colon tissue from a younger adult study group due to restricted availability. Thus, we relied on comparisons of tissues from babies to middle age and very old donors making a clear distinction between “normal” postnatal maturation -including cellular reorganization- and pathophysiological changes of ENS structure difficult. Therefore, interpretation need to be specified in broader studies including a younger adult study group and 3D morphometric data in correlation with functional readout. However, our data are mainly in line with previously published data describing alterations within the ENS during the process of aging and for the first time included also sodium channels, which potentially can cause alterations in neurotransmission and gut function. Sodium channels might present novel targets to develop novel cell- or drug-based therapies for the treatment of various neurogastroenterological diseases.

### Conflict of interest statement

The authors declare that the research was conducted in the absence of any commercial or financial relationships that could be construed as a potential conflict of interest.
